# Sex Dimorphism in Outcome of Trauma Patients Presenting with Severe Shock: A Multicenter Cohort Study

**DOI:** 10.3390/jcm12113701

**Published:** 2023-05-26

**Authors:** Stefan F. Van Wonderen, Merel Pape, Wietse P. Zuidema, Michael J. R. Edwards, Michael H. J. Verhofstad, Tjarda N. Tromp, Esther M. M. Van Lieshout, Frank W. Bloemers, Leo M. G. Geeraedts

**Affiliations:** 1 Department of Trauma Surgery, Amsterdam UMC location Vrije Universiteit Amsterdam, De Boelelaan 1117, 1081 HV Amsterdam, The Netherlands; 2 Department of Trauma Surgery, Radboud University Medical Center, 6525 GA Nijmegen, The Netherlands; 3 Trauma Research Unit, Department of Surgery, Erasmus MC, University Medical Center Rotterdam, 3015 GD Rotterdam, The Netherlands

**Keywords:** sex dimorphism, shock, hemorrhage, trauma, mortality, ICU, AKI, packed red blood cells

## Abstract

**Background**: The objective of this study was to determine the association between sex and outcome among severely injured patients who were admitted in severe shock. **Methods:** A retrospective multicenter study was performed in trauma patients (Injury Severity Score (ISS) ≥ 16) aged ≥ 16 presenting with severe shock (Shock Index > 1.3) over a 4-year period. To determine if sex was associated with mortality, Intensive Care Unit (ICU) admission, mechanical ventilation, blood transfusion and in-hospital complications, multivariable logistic regressions were performed. **Results:** In total, 189 patients were admitted to the Emergency Department in severe shock. Multivariable logistic regression analysis showed that female sex was independently associated with a decreased likelihood of acute kidney injury (OR 0.184; 95% CI 0.041–0.823; *p* = 0.041) compared to the male sex. A significant association between female sex and mortality, ICU admission, mechanical ventilation, other complications and packed red blood cells transfusion after admission could not be confirmed. **Conclusion:** Female trauma patients in severe shock were significantly less likely to develop AKI during hospital stay. These results could suggest that female trauma patients may manifest a better-preserved physiologic response to severe shock when compared to their male counterparts. Prospective studies with a larger study population are warranted.

## 1. Introduction

Globally, trauma-hemorrhage has a mortality rate of 1.9 million per year and affects a significant number of young people, resulting in nearly 75 million years of life lost [1,2]. Furthermore, survivors of trauma-hemorrhage have a poor functional status and significantly increased long-term mortality [3,4,5]. Shock in trauma is most often the result of hemorrhage. Massive hemorrhage is responsible for approximately 30–40% of trauma mortality and is recognized as the leading cause of preventable death. The treatment of bleeding patients is aimed at three main goals: stopping the bleeding, permissive hypotension if indicated and restoring blood volume during hemostatic resuscitation [6].

It appears that the female sex may have a better outcome after trauma-hemorrhage, multi-organ failure (MOF) and sepsis when compared to the male sex. Sex differences in trauma were reported for the first time in 1975, where the male sex was more prone to develop posttraumatic infections (7). Since then, studies have indicated the male sex as a major risk factor for sepsis, acute kidney injury (AKI) and MOF after trauma-hemorrhage [7,8,9,10,11,12,13,14,15,16,17]. In addition, clinical studies have also shown that female patients receive less blood products and have a lower mortality rate after trauma-associated shock [18,19,20]. However, previous studies had different parameters as indicators of inadequate tissue perfusion and shock, such as lactate levels or a systolic blood pressure (SBP) ≤ 89 mmHg [18,19].

Actual protocols in trauma-hemorrhage do not differentiate between sexes. However, if there is a significant difference in outcome, protocols may be changed and further research on the role of sex in trauma-hemorrhage may be of interest. In addition, previous studies with regard to sex dimorphism and trauma-hemorrhage did not focus specifically on patients in severe shock. Therefore, a retrospective multicenter study was performed to determine the association between sex and outcome among severe trauma patients presenting with severe shock. The hypothesis was that female sex is associated with a better-preserved physiologic response to severe shock (fewer ICU admissions, shorter ICU length of stay [LOS], packed red blood cells [pRBCs]) as well as lower mortality rates and lower inpatient complication rates (less mechanical ventilation, less MOF, less AKI and fewer wound infections) when compared to the male sex.

## 2. Materials and Methods

### 2.1. Study Design and Population

A retrospective study was performed in three Level 1 trauma centers between 1 January 2015 and 31 December 2018. Included were all trauma patients aged 16 years or older with an Injury Severity Score (ISS) ≥ 16 who were admitted to the Emergency Department (ED) in severe shock as defined by a Shock Index (SI) > 1.3. The SI is calculated by dividing the heart rate by the systolic blood pressure. This parameter was included since it was demonstrated that an initial SI > 1.3 at presentation to the ED was useful in predicting the likelihood of hospital admission and inpatient mortality whereas an SBP ≤ 89 mmHg may underestimate the effect of hemorrhagic shock on mortality and ICU admission [21]. Patients with drowning, asphyxia or burns, were excluded to reduce some heterogeneity within the population and because of the difference in pathophysiology. The study was exempted by the independent Medical Research Ethics Committees of the three participating hospitals.

### 2.2. Data Collection and Parameter Outcome

Demographic, injury and outcome parameters for all male and female patients were collected from the Trauma Registry. Injury severity was classified by the ISS and vital signs of the patients were scored with the Revised Trauma Score (RTS). ISS was used both as a continuous and as a categorical variable (16–24, 25–50 and 51–75) and RTS was used as a continuous and dichotomous variable (≤4). AIS of severe head, thoracic, abdominal and lower extremity trauma were dichotomized and defined as head AIS ≥ 3, thoracic AIS ≥ 3, abdominal AIS ≥ 3 and lower extremities AIS ≥ 3. SBP was collected both as a continuous and dichotomous variable (≤89). Base Excess (BE) was collected as a continuous variable. Glasgow coma scale (GCS) on scene and at admission were collected both as continuous and as dichotomous variables (≤8). Mechanism of injury (MOI) was either blunt or penetrating. Blunt injuries were defined as the result of direct contact of a blunt object with a body whereas penetrating trauma was defined as injury caused by stabbing or gunshot wounds. Moreover, prehospital cardiac arrest was collected as a dichotomous variable. In addition, pre-trauma comorbidity was collected as a dichotomous variable using the American Society of Anesthesiologists (ASA) Physical Status Classification System (healthy, ASA I, mild comorbidity, ASA II, and severe comorbidity, ASA ≥ III). Primary outcomes were mortality at 24 h, mortality until hospital discharge (MUHD) and ICU admission. Secondary outcomes were ICU LOS and mechanical ventilation in days, both as continuous variables. Other secondary outcomes were pRBCs in the first 24 h after admission as continuous and dichotomous variables (pRBCs in 24 h ≥ 1), mechanical ventilation during admission, AKI, wound infections and MOF as dichotomous variables. AKI was defined by a serum creatinine of >300 μmol/L or a urine production of <500 mL/day according to the Sequential Organ Failure Assessment (SOFA) score [22,23]. Wound infections were included as superficial wound infections, postoperative abscesses or osteomyelitis treated with antibiotics. MOF was defined by ≥3 points per organ for six different organs according to the SOFA score [23].

### 2.3. Statistical Analyses

Statistical tests were performed using IBM SPSS Statistics 27.0 and two-sided testing with a *p*-value < 0.05 set as statistically significant. A univariate data analysis was performed to compare patient demographics. Continuous variables were expressed as mean ± standard deviation (SD) and independent Student’s t-tests were used to compare means between two groups. Categorical variables were expressed as proportions and compared using Chi-squared (χ^2^) tests. In addition, median and interquartile ranges (IQR) were given for skewed distributions and a Mann–Whitney U-test was performed. Multivariable logistic regression analyses were performed to determine if sex was associated with mortality at 24 h, MUHD, ICU admission, ICU LOS, mechanical ventilation during admission, mechanical ventilation in days, MOF, AKI, wound infections and pRBCs in 24 h ≥ 1. Regression models were adjusted for a priori based covariates based on clinical relevance including age, ISS, RTS < 4, GCS ≤ 8 and comorbidity (≥ASA III). Before adjusting, predefined covariates were checked for effect modification by using an interaction term during logistic regression analyses. A maximum number of covariates was accepted during analysis powered to the smallest group in our primary outcome: mortality at 24 h. Missing data were not replaced and only complete cases were included in the multivariable models. Logistic regression analysis results will be presented as odds ratios (OR) with 95% confidence intervals (CI).

## 3. Results

### 3.1. Patients Characteristics

**Figure 1:**
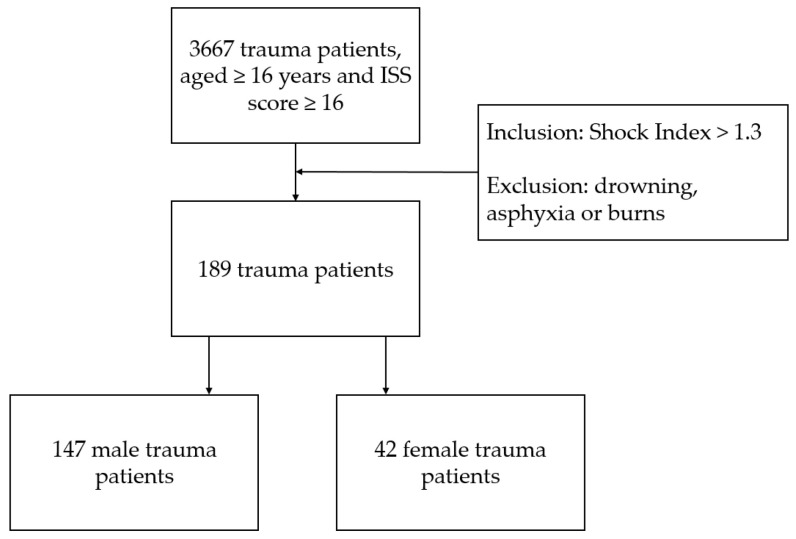
Flowchart of included trauma patients aged 16 years or older with an ISS score ≥ 16 and an SI > 1.3 without asphyxia, drowning or burn injury admitted to the ED of three Level 1 trauma centers.

During the 2015–2018 time period, 189 trauma patients aged 16 years or older with an ISS score ≥ 16 and an SI > 1.3 without asphyxia, drowning or burn injury were admitted to the ED of three Level 1 trauma centers (see Figure 1). The overall characteristics of the study population in severe shock are shown in Table 1. Females were more frequently presented with an ISS of 16–24 (18.4% vs. 33.3%, *p* = 0.038, Table 1) whereas males were more frequently admitted with an ISS of 25–50 (70.1% versus 50.0%, *p* = 0.016, Table 1). With regard to pretrauma comorbidity, females were more frequently healthy or had mild comorbidity (61.2% vs. 83.3%, *p* = 0.008, Table 1) whereas males had more frequently severe comorbidity (38.8% vs. 16.7%, *p* = 0.008, Table 1). In addition, median GCS was significantly lower in male patients when compared to female patients (3 vs. 4, *p* = 0.008, Table 1). Other patient characteristics were similar for both sexes.

### 3.2. Clinical Outcomes

In total, 94 patients who were admitted in severe shock at the ED died before hospital discharge (51.0% vs. 45.2%, *p* = 0.509, Table 2). No significant difference was found for mortality at 24 h (38.8% vs. 33.3%, *p* = 0.521, Table 2). There was no difference between male and female trauma patients with regard to ICU admission (83.0% vs. 76.2%, *p* = 0.317, Table 2) or mechanical ventilation during admission (70.7% vs. 61.9%, *p* = 0.275, Table 2). In addition, MOF (61.2% vs. 54.8%, *p* = 0.451, Table 2) and wound infections (8.8% vs. 11.9%, *p* = 0.556, Table 2) did not show a significant difference between sexes. Notably, AKI was significantly more present in the male sex compared to the female sex (19.7% vs. 4.8%, *p* = 0.021, Table 2). Neither median pRBCs (2 units for males (range 0–51) vs. 2 units for females (0–46), *p* = 0.564, Table 2) nor pRBCs in 24 h ≥ 1 unit (61.2% vs. 54.2%, *p* = 0.304, Table 2) showed a significant difference with regard to sex.

### 3.3. Multivariable Logistic Regression

Multivariable logistic regression analysis did not show a significant association between sex and mortality at 24 h (OR 1.453; 95% CI 0.571–3.695; *p* = 0.433; Table 3) or MUHD (OR 1.203; 95% CI 0.501–2.890; *p* = 0.679; Table 3). After adjusting for covariates, female sex was not associated with ICU admission (OR 0.684; 95% CI 0.280–1.671; *p* = 0.404) or mechanical ventilation during admission (OR 0.793; 95% CI 0.364–1.729 *p* = 0.560). With regard to inpatient complications during hospital admission, sex was not associated with MOF (OR 0.929; 95% CI 0.413–2.086; *p* = 0.858; Table 3) and wound infections (OR 1.424; 95% CI 0.455–4.454; *p* = 0.543; Table 3). In contrast, the female sex was independently associated with an 81.6% decreased likelihood of AKI when compared to the male sex (OR 0.184; 95% CI 0.041–0.823; *p* = 0.041; Table 3). With regard to blood transfusion, the female sex was not independently associated with decreased likelihood of receiving one or more pRBCs in the first 24 h after admission when compared to the male sex (OR 0.585; 95% CI 0.273–1.255; *p* = 0.169; Table 3).

## 4. Discussion

This study is performed in a contemporary population of severe trauma patients presenting with severe shock at Level 1 trauma centers in a mature trauma system with complete datasets. According to our knowledge, this is the first study with regard to sex dimorphism in trauma patients with severe shock as defined by SI > 1.3. In this study, female sex was significantly associated with an 81.2% decreased likelihood of AKI when compared to the male sex. On the contrary, a significant association between female sex and mortality at 24 h, MUHD, ICU admission, mechanical ventilation during admission, wound infections, MOF or pRBC transfusion in the first 24 h after admission, after adjusting for relevant covariates, could not be confirmed.

Standard definitions were used to obtain a homogeneous study population with minimal variations. In this study, ISS, AIS and RTS were used as surrogates for injury severity to cope with the heterogeneity of the trauma patient population. To identify the severity of hypovolemic shock in trauma patients, Allgower and Burri introduced the SI in 1967, which is the ratio of heart rate and SBP with a normal range of 0.5 to 0.7 in healthy persons [24]. With regard to severe shock, Al Jalbout et al. demonstrated that an SI > 1.3 was associated with a clinically significant increase in both the likelihood of hospital admission and inpatient mortality [21]. Therefore, an SI > 1.3 was used as surrogate for severe shock to evaluate the effect of hemorrhagic shock on mortality, ICU admission, inpatient complications and blood transfusion. Including an SI > 1.3 may also result in excluding other types of shock, e.g., neurogenic shock, since neurogenic shock results in a reduction in both heart rate and SBP.

In contrast to our findings, a retrospective analysis of 48,394 patients found a significantly lower mortality rate for females than for males after trauma-associated shock. In this study, shock was defined as SBP ≤ 89 mmHg whereas our study population included only patients in severe shock [19]. According to previous preclinical studies, the hypothesis of this study was that hormonally active female trauma patients have better outcomes after trauma-associated shock when compared to their male counterparts. The significantly lower mortality rate was especially found in females aged between 13 and 64 years, which was a surrogate for hormonally active females. This outcome difference was not found in preadolescent children aged ≤12 years and individuals aged ≥65 years, where the effects of sex hormones are presumably absent or diminished [19]. Similarly, a prospective study of 5192 patients by Deitch et al. used lactate levels as an indicator of inadequate tissue perfusion and shock [18]. The hypothesis of the authors was that hormonally active females tolerate shock better than male patients. The authors found that females in the premenopausal (14–44 years) and perimenopausal (45–54 years) age groups had significantly lower serum lactate levels when compared to their male counterparts whereas lactate levels in the preadolescence (<14 years) and postmenopausal (>55 years) age groups did not show a significant difference between sexes [18]. Similar to our findings regarding pRBC transfusion, Deitch et al. and McCrum et al. showed that a similar percentage of females and males required blood transfusion during the first 24 h after trauma. In contrast, the authors found that female patients received fewer pRBC units during the first 24 h and during hospital stay when compared to male patients [18,20]. In subgroup analysis, women with major trauma only received significantly fewer blood products during their hospital stay but not within the first 24 h when compared to their male counterparts [18]. Notably, the authors defined major trauma as presence of (1) major abdominal injuries with AIS of 4 or 5, or (2) three injuries or penetrating injuries with major blood loss, ≥ 2 long bone fractures, complex pelvic fracture, flail chest and/or major vascular injury, or (3) ISS ≥ 26, or (4) three or more injuries in a single body region with AIS ≥ 3 [18].

The current results showed that, after adjusting for covariates, females were associated with a decreased likelihood of AKI. Normally, the kidneys receive up to 25% of the cardiac output. Due to sympathetic nervous system activation during severe shock, blood is diverted away from noncritical organs and tissues to preserve blood supply to vital organs such as the heart and brain. Thus, failure of the systematic circulating blood volume as a result of shock can have a profound impact on renal perfusion and induction of acute tubular necrosis [22]. This suggests that female patients may have a better earlier physiologic response to severe shock with regard to our results of AKI. Notably, we did not differentiate phenotypic variability of AKI. By the way of comparison, a large retrospective study including 935,402 trauma victims showed that 9281 (0.99%) patients developed severe AKI. Similar to our findings, the authors showed that male trauma patients were significantly more prone to develop AKI compared to female trauma patients [16]. Similarly, another retrospective study including 681,730 trauma patients demonstrated that women are less likely than men to develop AKI, which is in line with our results [17]. However, it should be noted that the authors included all patients with trauma resulting in hospital admission whereas we included only patients presenting with severe shock [16,17].

Both similarities and differences in mechanism and pattern of injury among male and female patients were found in our population. With regard to the anatomical region of injuries, head AIS ≥ 3, thoracic AIS ≥ 3, abdominal AIS ≥ 3 and lower extremities AIS ≥ 3 were similar for both sexes. In contrast, females were more frequently presented with an ISS of 16–24 whereas males were more frequently admitted with an ISS of 25–50, suggesting that male patients were more often injured in multiple anatomical regions or more severely injured in one anatomical region. Therefore, we attempted to adjust for these differences by including ISS and AIS. In addition, median GCS was significantly lower in male patients when compared to female patients, and male patients were also more frequently admitted with a GCS < 8 when compared to female patients, which could more often lead to ICU admission and mechanical ventilation as the result of prehospital intubation. However, ICU LOS and mechanical ventilation during admission did not show a significant association between sexes.

There are several limitations in this retrospective study. Females represent only 22% of the study population with 42 patients. Nevertheless, the same percentages of included female patients were seen in similar studies [18,20]. Regarding the hormone-based hypotheses of previous clinical studies, the Trauma Registry did not collect hormonal status upon arrival at the ED. Therefore, it would be interesting to include age-related subgroups in our population with severe shock after trauma, which would act as surrogates for hormonal status: premenopausal or hormonally active phase, in which estrogen levels are the highest, and perimenopausal phase and postmenopausal phase (aged 45 or older), in which hormonal status is unclear or most likely low. However, due to the relatively low number of included patients, a subgroup analysis of patients aged 16–44 years was not performed because of the lack of power. A distribution of age within the female cohort is presented in Figure S1. Additionally, because of the retrospective setting of the Trauma Registry, AKI was defined by a serum creatinine of >300 μmol/L or a urine production of <500 mL/day according to the SOFA score, whereas the use of the Kidney Disease Improving Global Outcomes (KDIGO) AKI definition would be more accurate by including an increase in serum creatinine of ≥0.3 mg/dL (≥26.5 µmol/L) within 48 h, an increase in serum creatinine to ≥1.5 times the baseline within the previous 7 days, or urine volume ≤0.5 mL/kg/h for 6 h [25].

Also, unknown confounders may remain. The lack of information on medication such as β-blockers, inotropes and vasopressors may influence SI and could be significant. The lack of information on the usage of anticoagulants or platelet aggregation inhibitors may influence the need for blood transfusion and may therefore be a confounder. Additionally, large volumes of crystalloid resuscitation have also shown to be of influence in the development of AKI. In addition, use of contraceptive or hormonal replacement therapy is known to influence the actual hormonal status and could be a possible confounder.

The current study may be underpowered to detect a statistically significant survival advantage rate for females. Previous studies used larger registries with over 48,000 trauma patients in shock but a different indicator of shock was included and severe shock was not examined [19]. In this way, our study is unique when compared to similar studies with regard to sexual dimorphism in shock. The number of included covariates in logistic regression analysis was focused on the primary outcome. Hence, it is conceivable that the secondary outcomes might be subject to overadjustment, thereby necessitating caution in their evaluation. In addition, it should be noted that this investigation adopts an exploratory approach, thereby precluding any conclusive assertion of causality.

This study provides some insights into the association between sex and physiological outcomes after severe shock. The current results show some support for the hypothesis that female trauma patients may manifest a better-preserved physiologic response to severe shock when compared to their male counterparts. In the future, more information is needed on sex-specific physiological responses after trauma-induced severe shock. Future research may focus on prospective studies in which actual hormonal status is measured upon admission at the ED and during hospitalization. This information will help to understand the differences in sex-related outcomes after trauma-induced severe shock and may improve patient-specific treatment.

## 5. Conclusions

Female sex in trauma patients in severe shock was independently associated with an 81.6% decreased likelihood of AKI when compared to the male sex. In this study, a significant association between female sex and mortality, ICU admission, mechanical ventilation, MOF, wound infections and pRBC transfusion in the first 24 h after admission was not found. The current results suggest that female patients may have a better physiologic response to severe shock in trauma than male patients. However, causality, pathophysiology and associations with, e.g., mortality need to be investigated in larger populations.

## Figures and Tables

**Table 1:** Patient characteristics in the study population of trauma patients in severe shock (SI > 1.3).

	Total (*n* = 189)	Male (*n* = 147)	Female (*n* = 42)	*p*-Value
*Age*				
Age (years) median (IQR)	46 (30–62)	45 (30–60)	51 (29–65)	0.107
Age 16–44 years	88 (46.6%)	73 (49.7%)	15 (35.7%)	0.110
Age 45+ years	101 (53.4%)	74 (50.3%)	27 (64.3%)	0.110
*Injury severity*				
AIS Head ≥ 3	96 (50.8%)	77 (52.4%)	19 (45.2%)	0.414
AIS Thorax ≥ 3	125 (66.1%)	98 (66.7%)	27 (64.3%)	0.774
AIS Abdomen ≥ 3	52 (27.5%)	38 (25.9%)	14 (33.3%)	0.338
AIS Lower extremities ≥ 3	73 (38.6%)	56 (38.1%)	17 (40.5%)	0.780
ISS median (IQR)	34 (25–43)	34 (25–43)	29 (22–42)	0.265
ISS 16–24	41 (21.7%)	27 (18.4%)	14 (33.3%)	0.038
ISS 25–50	124 (65.6%)	103 (70.1%)	21 (50.0%)	0.016
ISS 51–75	23 (12.2%)	16 (10.9%)	7 (16.7%)	0.312
*Vital signs, laboratory results and physiologic scoring system*				
RTS median (IQR)	3.4 (2.6–6.4)	3.4 (2.6–6.2)	4.4 (3.2–7.0)	0.091
RTS < 4	85 (45.0%)	70 (47.6%)	15 (35.7%)	0.171
SBP mean ± SD (mmHg)	77 ± 16.13	76 ± 16.67	79 ± 13.96	0.244
SBP ≤ 89 mmHg	148 (78.3%)	116 (78.9%)	32 (76.2%)	0.706
Shock Index median (IQR)	1.6 (1.4–1.8)	1.6 (1.4–1.9)	1.5 (1.4–1.8)	0.236
Base Excess median (IQR)	−9.0 (−15.0—5.0)	−9.0 (−15.4–5.0)	−9.3 (−13.8—4.2)	0.947
*Neurological score*				
GCS on scene median (IQR)	5 (3–14)	5 (3–14)	3 (3–14)	0.836
GCS at admission median (IQR)	3 (3–11)	3 (3–8)	4 (3–13)	0.008
GCS ≤ 8 at admission	127 (67.2%)	104 (70.7%)	23 (54.8%)	0.052
*Prehospital*				
Blunt	165 (87.3%)	127 (86.4%)	38 (90.5%)	0.484
Penetrating	24 (12.7%)	20 (13.6%)	4 (9.5%)	0.484
Cardiac arrest	17 (20.2%)	13 (20.0%)	4 (21.1%)	1.000
Prehospital intubation	125 (67.6%)	103 (71.0%)	22 (55.0%)	0.055
P-HEMS	90 (47.6%)	71 (48.3%)	19 (45.2%)	0.726
*Comorbidity*				
Healthy or mild (ASA I or II)	125 (66.1%)	90 (61.2%)	35 (83.3%)	0.008
Severe comorbidity (≥ASA III)	64 (33.9%)	57 (38.8%)	7 (16.7%)	0.008

AIS, Abbreviated Injury Scale; ASA, American Society of Anesthesiologists; ED, Emergency Department; GCS, Glasgow coma scale; ISS, Injury Severity Score; IQR, Interquartile Range; P-HEMS, physician-staffed helicopter emergency medical services attendance; RTS, Revised Trauma Score; SD, standard deviation; SBP, systolic blood pressure.

**Table 2:** Comparison by sex of clinical outcomes of trauma patients in severe shock (SI > 1.3).

	Total (*n =* 189)	Male (*n =* 147)	Female (*n =* 42)	*p*-Value
*Mortality*				
Mortality at 24 h	71 (37.6%)	57 (38.8%)	14 (33.3%)	0.521
Mortality until hospital discharge	94 (49.7%)	75 (51.0%)	19 (45.2%)	0.509
*ICU outcomes*				
ICU admission	154 (81.5%)	122 (83.0%)	32 (76.2%)	0.317
ICU days median (IQR)	4 (2–12)	4 (2–12)	5 (2–12)	0.642
MV during admission	130 (68.8%)	104 (70.7%)	26 (61.9%)	0.275
MV duration in days median (IQR)	3 (1–9)	3 (1–9)	2 (1–10)	0.568
*Complications*				
Multiple organ failure	113 (59.8%)	90 (61.2%)	23 (54.8%)	0.451
Acute kidney injury	31 (16.4%)	29 (19.7%)	2 (4.8%)	0.021
Wound infection	18 (9.5%)	13 (8.8%)	5 (11.9%)	0.556
*Transfusion*				
pRBCs in 24 h ≥ 1	112 (59.3%)	90 (61.2%)	22 (54.2%)	0.304
pRBCs in 24 h median (IQR)	2 (0–5)	2 (0–5)	2 (0–5)	0.711

ICU, Intensive Care Unit; IQR, Interquartile Range; LOS, length of stay; MV, mechanical ventilation; pRBCs, packed red blood cells.

**Table 3:** The association of female sex with clinical outcome parameters in trauma patients presenting with severe shock (SI > 1.3).

	OR	95% CI	*p*-Value
*Mortality*			
Mortality at 24 h	1.453	0.571–3.695	0.433
Mortality until hospital discharge	1.203	0.501–2.890	0.679
*ICU outcomes*			
ICU admission	0.684	0.280–1.671	0.404
Mechanical ventilation during admission	0.793	0.364–1.729	0.560
*Complications*			
Multiple organ failure	0.929	0.413–2.086	0.858
Acute kidney injury	0.184	0.041–0.823	0.041
Wound infection	1.424	0.455–4.454	0.543
*Transfusion*			
pRBCs in 24 h ≥ 1	0.585	0.273–1.255	0.169

CI, Confidence interval; ICU, Intensive Care Unit; LOS, length of stay; pRBCs, packed red blood cells. Adjusted for: age, ISS, RTS < 4, GCS ≤ 8 and comorbidity (≥ASA III) (see Appendix A).

## Data Availability

The datasets generated and analyzed during the current study are available from the corresponding authors on reasonable request.

## Supplementary Materials

The following supporting information can be downloaded at: https://www.mdpi.com/article/10.3390/jcm12113701/s1, Figure S1. Distribution of female sex regarding age.

## Appendix A

**Table A1:** Multivariable logistic regression analysis of mortality at 24 h in trauma patients presenting with severe shock.

Mortality at 24 h								
	B	S.E.	Wald	df	Sig.	Exp(B)	95% C.I. for EXP(B)	
							Lower	Upper
Sex	0.373	0.476	0.615	1	0.433	1.453	0.571	3.695
ISS	0.071	0.016	18.993	1	0.000	1.074	1.040	1.108
Age	0.022	0.010	5.030	1	0.025	1.023	1.003	1.043
RTS < 4	0.992	0.410	5.854	1	0.016	2.696	1.207	6.019
GCS ≤ 8	1.369	0.544	6.339	1	0.012	3.931	1.354	11.408
Severe comorbidity (≥ASA III)	1.275	0.411	9.647	1	0.002	3.580	1.601	8.007
Constant	−6.262	1.026	37.244	1	0.000	0.002		

**Table A2:** Multivariable logistic regression analysis of mortality until hospital discharge in trauma patients presenting with severe shock.

Mortality Until Hospital Discharge								
	B	S.E.	Wald	df	Sig.	Exp(B)	95% C.I. for EXP(B)	
							Lower	Upper
Sex	0.185	0.447	0.171	1	0.679	1.203	0.501	2.890
ISS	0.060	0.016	13.764	1	0.000	1.061	1.028	1.095
Age	0.037	0.011	12.683	1	0.000	1.038	1.017	1.060
RTS < 4	0.797	0.410	3.788	1	0.052	2.219	0.994	4.954
GCS ≤ 8	1.427	0.476	9.003	1	0.003	4.165	1.640	10.579
Severe comorbidity (≥ASA III)	1.243	0.406	9.375	1	0.002	3.467	1.564	7.686
Constant	−5.667	0.959	34.947	1	0.000	0.003		

**Table A3:** Multivariable logistic regression analysis of ICU admission in trauma patients presenting with severe shock.

ICU Admission								
	B	S.E.	Wald	df	Sig.	Exp(B)	95% C.I. for EXP(B)	
							Lower	Upper
Sex	−0.380	0.456	0.695	1	0.404	0.684	0.280	1.671
ISS	−0.012	0.014	0.729	1	0.393	0.988	0.961	1.016
Age	−0.022	0.010	4.916	1	0.027	0.979	0.960	0.997
RTS < 4	−0.109	0.464	0.055	1	0.815	0.897	0.361	2.229
GCS ≤ 8	0.532	0.500	1.132	1	0.287	1.702	0.639	4.529
Severe comorbidity (≥ASA III)	−0.354	0.424	0.699	1	0.403	0.702	0.306	1.611
Constant	2.928	0.775	14.271	1	0.000	18.694		

**Table A4:** Multivariable logistic regression analysis of mechanical ventilation during admission in trauma patients presenting with severe shock.

Mechanical Ventilation during Admission								
	B	S.E.	Wald	df	Sig.	Exp(B)	95% C.I. for EXP(B)	
							Lower	Upper
Sex	−0.231	0.397	0.339	1	0.560	0.793	0.364	1.729
ISS	−0.003	0.012	0.061	1	0.806	0.997	0.973	1.022
Age	−0.017	0.008	4.331	1	0.037	0.983	0.967	0.999
RTS < 4	−0.511	0.412	1.543	1	0.214	0.600	0.268	1.344
GCS ≤ 8	1.238	0.436	8.078	1	0.004	3.448	1.468	8.096
Severe comorbidity (≥ASA III)	−0.094	0.371	0.064	1	0.801	0.910	0.440	1.885
Constant	1.266	0.631	4.031	1	0.045	3.548		

**Table A5:** Multivariable logistic regression analysis of multiple organ failure in trauma patients presenting with severe shock.

Multiple Organ Failure								
	B	S.E.	Wald	df	Sig.	Exp(B)	95% C.I. for EXP(B)	
							Lower	Upper
Sex	−0.074	0.413	0.032	1	0.858	0.929	0.413	2.086
ISS	0.047	0.015	9.854	1	0.002	1.048	1.018	1.079
Age	0.032	0.010	11.424	1	0.001	1.033	1.014	1.052
RTS < 4	0.509	0.399	1.628	1	0.202	1.663	0.761	3.632
GCS ≤ 8	0.838	0.420	3.982	1	0.046	2.312	1.015	5.268
Severe comorbidity (≥ASA III)	0.688	0.388	3.139	1	0.076	1.989	0.930	4.257
Constant	−3.646	0.776	22.056	1	0.000	0.026		

**Table A6:** Multivariable logistic regression analysis of acute kidney injury in trauma patients presenting with severe shock.

Acute Kidney Injury								
	B	S.E.	Wald	df	Sig.	Exp(B)	95% C.I. for EXP(B)	
							Lower	Upper
Sex	−1.693	0.765	4.902	1	0.027	0.184	0.041	0.823
ISS	0.000	0.015	0.000	1	0.988	1.000	0.970	1.030
Age	0.006	0.010	0.293	1	0.588	1.006	0.986	1.026
RTS < 4	−0.354	0.476	0.555	1	0.456	0.702	0.276	1.783
GCS ≤ 8	−0.082	0.514	0.025	1	0.874	0.922	0.336	2.526
Severe comorbidity (≥ASA III)	−0.080	0.452	0.031	1	0.860	0.923	0.381	2.240
Constant	−1.413	0.764	3.417	1	0.065	0.244		

**Table A7:** Multivariable logistic regression analysis of wound infection in trauma patients presenting with severe shock.

Wound Infection								
	B	S.E.	Wald	df	Sig.	Exp(B)	95% C.I. for EXP(B)	
							Lower	Upper
Sex	0.354	0.582	0.369	1	0.543	1.424	0.455	4.454
ISS	0.003	0.019	0.025	1	0.873	1.003	0.966	1.041
Age	−0.014	0.013	1.060	1	0.303	0.986	0.961	1.013
RTS < 4	−0.012	0.615	0.000	1	0.985	0.988	0.296	3.301
GCS ≤ 8	−0.605	0.627	0.929	1	0.335	0.546	0.160	1.868
Severe comorbidity (≥ASA III)	0.274	0.570	0.231	1	0.631	1.315	0.431	4.017
Constant	−1.528	0.935	2.675	1	0.102	0.217		

**Table A8:** Multivariable logistic regression analysis of one or more pRBCs in 24 in trauma patients presenting with severe shock.

pRBCs in 24 h ≥ 1								
	B	S.E.	Wald	df	Sig.	Exp(B)	95% C.I. for EXP(B)	
							Lower	Upper
Sex	−0.536	0.389	1.895	1	0.169	0.585	0.273	1.255
ISS	0.019	0.012	2.565	1	0.109	1.019	0.996	1.044
Age	−0.018	0.008	4.731	1	0.030	0.983	0.967	0.998
RTS < 4	−1.025	0.373	7.575	1	0.006	0.359	0.173	0.744
GCS ≤ 8	−0.360	0.420	0.736	1	0.391	0.697	0.306	1.588
Severe comorbidity (≥ASA III)	−0.254	0.352	0.521	1	0.470	0.776	0.389	1.546
Constant	1.477	0.627	5.555	1	0.018	4.382		
