# Supplementary figures and images for: Sex Dimorphism in Outcome of Trauma Patients Presenting with Severe Shock: A Multicenter Cohort Study
Source: J Clin Med. 2023 May 26;12(11):3701. doi: 10.3390/jcm12113701 (PMC10253981; doi:10.3390/jcm12113701)

## Supplemental Figure

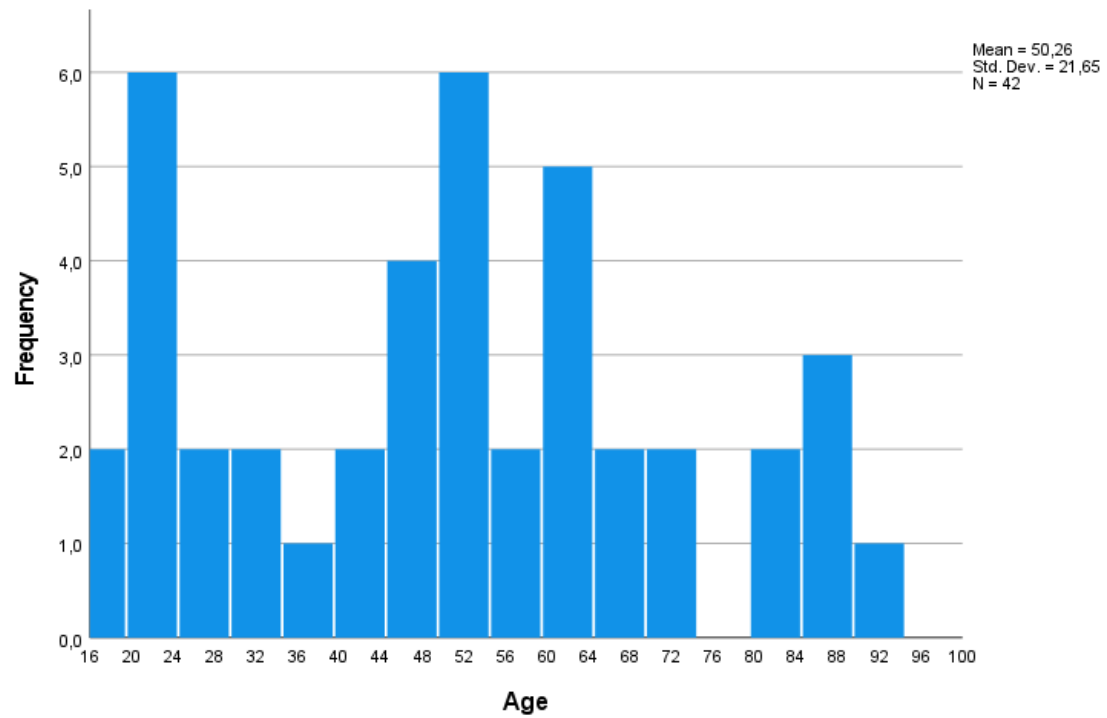

Figure S1. Distribution of female sex regarding age.

Supplement: Supplementary file 1 [file jcm-12-03701-s001.zip › jcm-2390192-supplementary.pdf]
